# Fluoride Retention in Root Dentin following Surface Coating Material Application

**DOI:** 10.3390/jfb14030171

**Published:** 2023-03-22

**Authors:** Katsushi Okuyama, Yasuhiro Matsuda, Hiroko Yamamoto, Kohtaku Suzuki, Kohei Shintani, Takashi Saito, Mikako Hayashi, Yukimichi Tamaki

**Affiliations:** 1 Department of Dental Material Science, Division of Oral Functional Sciences and Rehabilitation, Asahi University School of Dentistry, Mizuho 501-0296, Japan; 2 Division of Clinical Cariology and Endodontology, Department of Oral Rehabilitation, School of Dentistry, Health Sciences University of Hokkaido, Tobetsu 061-0293, Japan; 3 Department of Restorative Dentistry and Endodontology, Osaka University Graduate School of Dentistry, Suita 501-0296, Japan; 4 The Wakasa Wan Energy Research Center, Tsuruga 914-0192, Japan

**Keywords:** in-air micro-PIXE/PIGE, tooth-bound fluoride, KOH treatment, fluoride-containing coating material, root dentin, hydroxyapatite

## Abstract

This study aimed to use an in-air micro-particle-induced X-ray/gamma emission (in-air µPIXE/PIGE) system to evaluate tooth-bound fluoride (T-F) in dentin following the application of fluoride-containing tooth-coating materials. Three fluoride-containing coating materials (PRG Barrier Coat, Clinpro XT varnish, and Fuji IX EXTRA) and a control were applied to the root dentin surface of human molars (n = 6, total 48 samples). Samples were stored in a remineralizing solution (pH 7.0) for 7 or 28 days and then sectioned into two adjacent slices. One slice of each sample was immersed in 1M potassium hydroxide (KOH) solution for 24 h and rinsed with water for 5 min for the T-F analysis. The other slice did not undergo KOH treatment and was used to analyze the total fluoride content (W-F). The fluoride and calcium distributions were measured in all the slices using an in-air µPIXE/PIGE. Additionally, the amount of fluoride released from each material was measured. Clinpro XT varnish demonstrated the highest fluoride release among all the materials and tended to show high W-F and T-F and lower T-F/W-F ratios. Our study demonstrates that a high fluoride-releasing material shows high fluoride distribution into the tooth structure and low conversion from fluoride uptake by tooth-bound fluoride.

## 1. Introduction

Dentin hypersensitivity is a common, transient oral pain condition that results immediately upon stimulation of exposed dentine and resolves upon stimulus removal [1]. Dentin hypersensitivity can be managed by several methods. One of the methods involves occluding the dentin tubules to block the stimulus. Certain crystals composed of calcium from the dentin tubules, and some ions (e.g., fluoride and phosphate), are precipitated into the dentin tubules. Numerous active principles of tubular occlusion have been described, including chemical: fluorides [2], oxalates [3], or arginine [4], and physical: adhesives [5,6] or laser therapies [7]. In addition, certain dental materials (resin composites, glass ionomer cements, and bonding agents) cover the root surface and seal the open dentin tubules. In many cases, fluoride-containing coating materials are applied to the enamel or dentin surfaces for caries prevention and inhibition of hypersensitivity, since fluoride makes the tooth structure harder, and fluoride crystals (products) are filled into the dentin tubules to produce a barrier obstructing certain stimuli.

When fluoride uptake occurs at the tooth surface, it is converted into two kinds of fluoride products. The first is a calcium fluoride-like (CaF_2_-like) material [8,9,10]. This product could serve as a fluoride provider to the tooth structure and helps in decreasing tooth demineralization. The CaF_2_-like material is loosely bound to the tooth structure and can be eliminated using potassium hydroxide (KOH). Hence, it is also known as KOH-soluble fluoride (S-F). The other product is a fluorapatite-like product. This product is converted from hydroxyapatite (HAP) because the hydroxyl ion is replaced with a fluoride ion and is firmly bound and insoluble in KOH (tooth-bound fluoride: T-F) [11]. This fluorapatite-like product has lower solubility in acid than HAP; therefore, the tooth structure is harder and caries resistant. T-F also contributes to caries prevention [12]. Studies have demonstrated S-F and T-F on/in tooth structures following topical fluoride application to the tooth surface [8,13,14,15,16,17,18,19,20,21]. Most previous studies have demonstrated that T-F can be measured on dissolved enamel or dentin after KOH treatment. In some previous studies [13,16,20], tooth samples were stored in hydrochloric acid (HCl) or perchloric acid (HClO_4_) solution for dissolution, and then the concentration of fluoride in the solution was measured. Therefore, tooth samples were destroyed and could not be measured repeatedly. In other studies, several tooth samples were collected by grinding the tooth surface of a certain thickness and then the collected sample was dissolved [8,17,21], or a small amount of HCl or HClO_4_ was dropped onto the tooth surface and then the treated solution was collected [18,19]. After that, the concentration of fluoride in the solution was measured. This procedure was performed two or three times. From this method, the amount of T-F in each layer (each acid drop) was found and compared between each layer (about 50 µm each). However, the fluoride value could not be continuously measured. 

Komatsu et al. [22] measured and analyzed the T-F distribution in the enamel at the cavity wall and dentin at the cavity floor from restorative (filling) materials, and Okuyama et al. [23] reported the distribution of T-F, which is derived from the tooth-coating materials on the enamel surface, using an in-air micro-particle-induced X-ray/gamma emission (in-air µPIXE/PIGE) system. The PIXE/PIGE system is one of the methods used to detect the distribution of elements on a surface. This method uses particle-induced X-ray emission (PIXE) or particle-induced gamma-ray emission (PIGE). The tested samples are set in air, and one can measure the distribution of elements without sample damage; thus, the same sample can be measured several times, unlike measurements by energy dispersive X-ray (EDX) [24,25] or electron probe micro-analyzer (EPMA) [26,27,28], where the sample for these analyses needs to be coated with metal or carbon by spattering for observation or measurement. In our PIXE/PIGE analysis, the tooth specimen did not perish, and the T-F was continuously measured from the superficial surface to the deep area.

However, no study exists on the distribution of T-F in the root dentin. The structure of root dentin could be different from that of enamel or coronal dentin (e.g., tubule occlusion and dentin quality). Furthermore, the conversion rate of T-F to the total fluoride (whole fluoride: W-F) is unclear. Fluoride incorporated into the tooth minerals from dental materials enhances the acid resistance of tooth structures. Although fluoride from materials directly penetrates into the tooth structure, whether fluoride is completely incorporated into the tooth minerals remains unknown. It would be important to know how much of the fluoride uptake is converted at the various deep levels, which could result in the development of new caries prevention methods.

This study aimed to evaluate dentin-bound fluoride derived from various fluoride-containing tooth-coating materials using an in-air micro-PIXE/PIGE system. The null hypothesis is that a material that releases a lot of fluoride will result in a large amount of tooth-bound fluoride.

## 2. Materials and Methods

### 2.1. Fluoride Distribution in Root Dentin following the Application of Coating Materials

**Figure 1:**
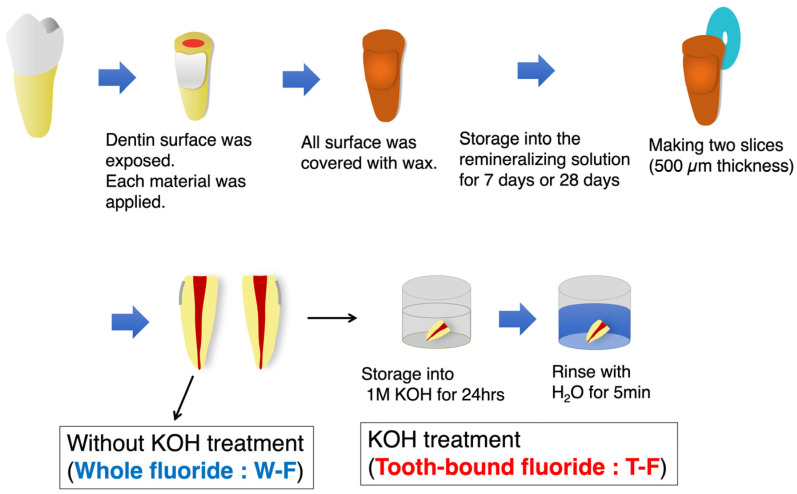
Outline of the experimental procedure. The teeth were separated from their crown, and the root dentin surface was exposed. Coating materials were subsequently applied to the dentin surface, and the whole surface was covered with wax. Each specimen was stored in remineralizing solution at 37 °C for 7 or 28 days. After storing the specimens, the samples were sectioned into two slices. Measurements of the fluoride and calcium concentrations were conducted using in-air µPIXE/PIGE. One slice was immersed in 50 mL 1M KOH solution for 24 h and subsequently rinsed with water for 5 min to analyze the tooth-bound fluoride (T-F). The sample that was not KOH treated was analyzed for total fluoride (whole fluoride: W-F).

Three different fluoride-containing coating materials were used: S-PRG filler-containing material (PB; PRG Barrier Coat; Shofu, Kyoto, Japan), fluoride-containing coating material (CX; Clinpro XT varnish; 3M, St. Paul, MN, USA), and glass ionomer cement (FE; Fuji IX EXTRA; GC, Tokyo, Japan). A non-fluoride-containing material (MB; Clearfil Mega Bond; Kuraray Noritake Dental, Tokyo, Japan) was used as the control material (Table 1). Forty-eight extracted human molars were used in the present study. Teeth were collected from the Asahi University Medical and Dental Center after obtaining informed consent from the patients. After collecting, teeth with caries or restoration were excluded. The collected teeth were cleaned and stored in 0.1% thymol solution at 4 °C to maintain hydration until use. The study protocol was approved by the Research Ethics Committee of Asahi University School of Dentistry (Approval Number: 30008). Figure 1 illustrates the procedure used in this study. The buccal or lingual root surface was ground using #600 SiC paper to expose the flat dentin surface. Each material was applied to the exposed root dentin surface of each tooth according to the manufacturer’s instructions. After applying the materials, all the surfaces of the specimens were coated with sticky wax. After coating, each specimen was stored in remineralizing solution (0.02 M HEPES, 1.8 mM KH_2_PO_4_, 3.0 mM CaCl_2_, 130 mM KCl; pH 7.0) [29,30,31] at 37 °C for 7 or 28 days. These storage periods were defined on the basis of the amount of fluoride released from fluoride-containing materials (mainly glass ionomer cement) [32,33,34]. From day 7, the amount of released fluoride was not large. From about day 28, the amount of fluoride released was stable. After maintaining the specimens according to the storage period, the specimens were sectioned into two slices (500-µm thickness) perpendicular to the long axis of the tooth using a low-speed diamond saw (Isomet; Buehler, Lake Bluff, IL, USA) under water irrigation. Fluoride and calcium concentrations were measured in two adjacent slices. One slice was immersed in 50 mL of 1M KOH solution for 24 h and then rinsed with water for 5 min to remove S-F according to previous studies [8,10] and analyze the T-F. The other slice was not subjected to KOH treatment and was used for W–F analysis. 

**Figure 2:**
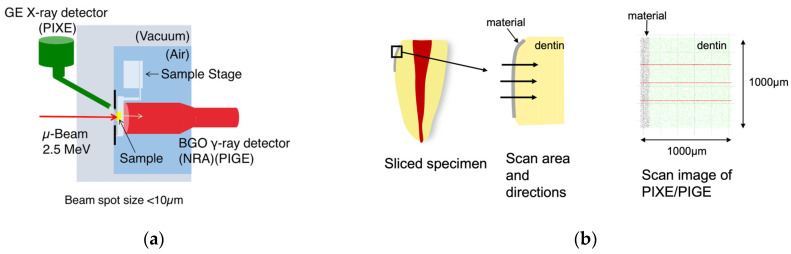

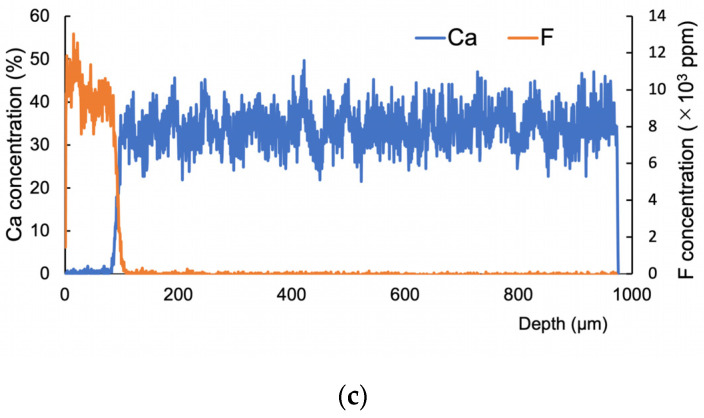
Schematic representations of the external microbeam apparatus for µPIGE/PIXE measurements. (**a**) Schematic drawing of the external microbeam apparatus for the µPIXE/PIGE system. (**b**) Schematic outline of the PIXE/PIGE analysis. The analysis area is shown in the left and middle images. The left image shows a cross-section view of the sliced tooth specimen. The black box indicates a scan area on this analysis. The middle image shows an enlarged view of the scan area. Horizontal arrows indicate the positions of the line analysis. The right image shows the area of the analysis and positions of the line analysis. Scan area is 1000 µm × 1000 µm square. (**c**) Typical profile indicating the concentrations of Ca and F.

The fluoride and calcium distribution in the specimens were evaluated using an in-air µPIXE/PIGE system at the Wakasa Wan Energy Research Center, as previously described [22,23,35]. Briefly, a 2.5-MeV proton beam was extracted through a thin silicon nitride window into air and used to irradiate a sample mounted on a sample stage set in air. The beam spot had a diameter of less than 10 µm. A nuclear reaction (that is, ^19^F (p, αγ) ^16^O) was initiated, and gamma rays were induced to measure the fluoride content. The calcium content was measured using PIXE (Figure 2a). Fluoride and calcium concentrations were measured in 1000-µm × 1000-µm square areas by scanning along three analysis lines at arbitrary positions in the scanned area (Figure 2b,c).

To evaluate the fluoride distribution, the average calcium concentration in each specimen was calculated at approximately 10 µm intervals to a depth of 500 µm from the superficial surface. The dentin–material interface is defined as the layer containing 90% of the calcium concentration found in intact dentin [31,36]. Fluoride concentration was also calculated at approximately 20 µm intervals from the defined surface, similar to the calcium measurement. The cumulative fluoride concentration (parts per million (ppm)) in each specimen was calculated from the dentin–material interface to a depth of 100 µm to that surface. The ratio of T-F/W-F for each specimen was also calculated.

### 2.2. Measurement of KOH-Insoluble and Whole Fluoride in Fluoride-Treated HAP

To create fluoride-treated HAP (f-HAP), 400 mg of HAP powder (Kishida Chemical, Osaka, Japan) was immersed in a plastic tube with 15 mL of remineralizing solution (same as previously described; pH 7.0). The fluoride concentration of the solution was adjusted to 1, 10, or 100 ppm using NaF, and the sample was shaken for 7 or 28 days at room temperature. After storage, the samples were centrifuged at 3000 rpm for 5 min (Model 6200; Kubota Co., Tokyo, Japan). The residues were collected, rinsed twice with distilled deionized water (DDW), filtered, and then dried at 100 °C for 2 h to yield f-HAP. 

KOH treatment of f-HAP was performed using modified procedures on the basis of previous reports [8,13,37]. First, 200 mg of f-HAP was placed into plastic tubes with 10 mL of 1M KOH solution. The tubes were then shaken at room temperature for 24 h. The samples were subsequently centrifuged at 3000 rpm for 5 min, after which the supernatant (KOH) and residue (k-HAP) were separated.

The procedure for measuring W-F or T-F in f-HAP was modified on the basis of previous reports [8,16]. f-HAP and k-HAP were rinsed with 100 mL DDW for 5 min, washed twice with centrifugation (3000 rpm for 5 min), and then dried (100 °C for 2 h). Twenty milligrams of f-HAP or k-HAP was added to 1.5 mL of 1M HCl and maintained at room temperature for 24 h. Thereafter, 0.3 mL of the sample was added to 2.7 mL of 15% CH_3_COONa for neutralization and buffered with 0.3 mL of total ionic strength adjustment buffer (TISAB III; Thermo Fisher Scientific, Chelmsford, MA, USA). Fluoride was analyzed using a fluoride electrode (Orion 9609BNWP, Thermo Fisher Scientific). The amounts of W-F and T-F were calculated (µmol of F/mg of f-HAP). In addition, the ratio of T-F/W-F for each HAP specimen was calculated.

### 2.3. Measurement of Fluoride Release from Materials 

Fluoride released from the materials into the water was measured as described previously [29]. Briefly, six disc-shaped specimens of each material (10-mm diameter and 1-mm thickness) were prepared. The specimens were stored in plastic beakers containing 8 mL DDW and incubated at 37 °C without shaking. After 1 day, the specimens were removed from the beakers, washed with 2 mL of DDW, and placed in 8 mL of fresh DDW. The DDW (8 mL) used for storage and 2 mL of DDW used for washing were collected to measure the fluoride concentration. This procedure was repeated on days 2, 3, and 7. After day 7, this procedure was repeated every seven days for 28 days. The collected solution was mixed with a 0.3 mL TISAB III buffer solution, and the fluoride concentration (ppm) was subsequently determined using a fluoride electrode. The amount of fluoride released was expressed in micrograms per square centimeter (µg/cm^2^).

### 2.4. Data Analysis

The obtained data on fluoride distribution according to PIXE/PIGE analysis were analyzed using the Steel–Dwass test comparison among materials, or Mann–Whitney U test for comparison of W-F and T-F in the same material and under the same conditions (*p* > 0.05). The values of W-F and T-F on HAP were analyzed using two-way analysis of variance (ANOVA) (concentration of solution vs. duration of exposure) and Tukey’s test (*p* > 0.05). The amount of fluoride released from the materials was analyzed using the Games–Howell test (*p* > 0.05). All the analyses were performed using R statistical analysis software ver. 4.2.2. (R Foundation for Statistical Computing, Vienna, Austria).

## 3. Results

### 3.1. Fluoride Distribution in the Tooth Structure

**Figure 3:**
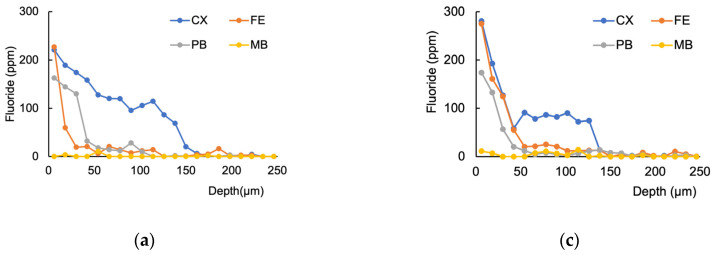

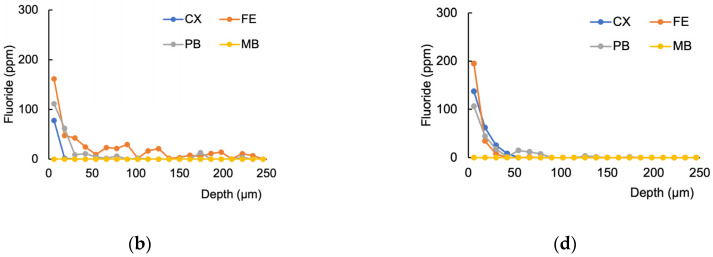
Fluoride (F) distribution in the root dentin from the interface between the material and dentin to a depth of 250 µm at day 7 and day 28; day 7 on total fluoride content (W-F) (**a**), day 7 on tooth-bound fluoride (T-F) (**b**), day 28 on W-F (**c**), and day 28 on T-F (**d**) are indicated. The fluoride concentration decreased from the interface to the deeper area.

**Figure 4:**
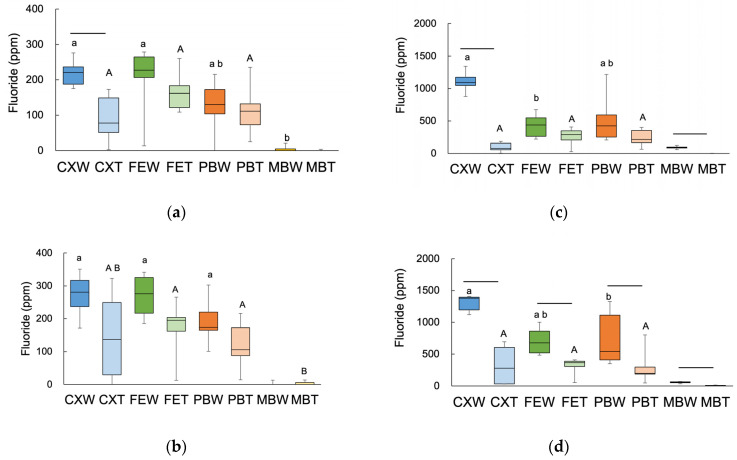
Amount of fluoride at the interface or the cumulative fluoride from the interface to the point of 100 µm from the interface; day 7 at the interface (**a**), day 28 at the interface (**b**), day 7 cumulative (**c**), and day 28 cumulative (**d**) are indicated. Bars connected values indicate a significant difference between the total fluoride content (W-F) and tooth-bound fluoride (T-F) on the same material (*p* < 0.05). The same small or large letters indicate no significant difference in the W-F or T-F among materials, respectively (*p* > 0.05).

The distribution of fluoride uptake from the materials into the root dentin is shown in Figure 3. The highest fluoride concentration was observed at the interface between dentin and the material; the fluoride concentration decreased over the first 100 µm from the surface. Figure 4 shows the amounts of W-F (W) and T-F (T) in the interface area and the cumulative value at a 100 µm depth. Upon comparison of W-F and T-F, the CX samples had more W-F than T-F at the interface area in the 7 days group. For cumulative fluoride, significant differences were observed between W-F and T-F with CX and MB at day 7 and with all the materials at day 28.

Upon comparing the materials, we found no significant difference among fluoride-containing materials (CX, FE, and PB) at the interface area at both periods and on the two kinds of fluoride. For cumulative fluoride, W-F was significantly different between CX and FE on day 7, or between CX and PB on day 28. There was no significant difference in the T-F between the fluoride-containing materials. MB showed the lowest value against fluoride-containing materials, except for W-F at the interface area at day 7 and T-F on the cumulative fluoride at day 28. For the T-F/W-F ratio (Table 2), CX demonstrated the lowest ratio of cumulative fluoride at day 7. Under these conditions, CX tended to exhibit a lower ratio than the other materials.

### 3.2. W-F and T-F in Fluoride-Treated HAP

Table 3 shows the amount of W-F and T-F and the ratio of T-F/W-F in f-HAP on days 7 and 28. According to the two-way ANOVA, as the concentration of solution increased, the amount of W-F or T-F also increased (*p* < 0.001). On W-F (fluoride in f-HAP), there was no significant difference between days 7 and 28 (*p* = 0.615), and there was no interaction between the concentration of the fluoride solution and the HAP exposure period (*p* = 0.263). In contrast, an interaction between the concentration and HAP exposure period was observed in T-F (*p* < 0.001). There was a significant difference between days 7 and 28 in the 100 ppmF exposure group. The W-F was higher than that of T-F under the same conditions, except for 1 ppm in the day 28 group, which showed no difference between the two fluorides. 

For the T-F/W-F ratio, the 100 ppm group showed a lower ratio than the other concentration groups in the 7 days duration period. Considering the duration period, the day 28 group had a lower ratio than the day 7 group at 1 ppm; however, at 100 ppm, the day 28 group had a higher ratio than the day 7 group. 

### 3.3. Fluoride Release from the Materials

**Figure 5:**
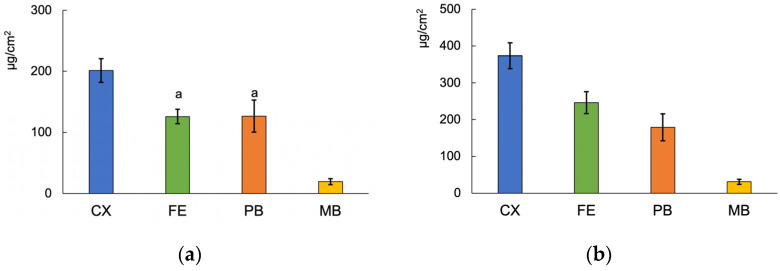
Cumulative fluoride release from the materials until 7 days (**a**), or 28 days (**b**). The same letter indicates no significant difference (*p* > 0.05).

The cumulative amounts of fluoride released from the materials over 7 and 28 days are shown in Figure 5. There was no significant difference in the fluoride release between the FE and PB groups after 7 days. CX and MB showed the highest and lowest fluoride release among all the materials at both periods, respectively.

## 4. Discussion

Both S-F and T-F are crucial for the prevention of caries. We expected that both S-F and T-F would be present after the application of fluoride-containing dental materials. Many studies have reported on S-F following fluoride application [8,9,10]. However, we focused on T-F, which could be detected by using an in-air micro-PIXE/PIGE system. In addition, we evaluated the amount of fluoride in f-HAP and the fluoride released from the materials for a more detailed analysis of the T-F relationship with fluoride.

We measured the T-F converted from material-released fluoride using an in-air µ PIXE/PIGE. Most previous studies [8,9,23,24,25] have demonstrated that T-F can be measured on dissolved enamel or dentin after KOH treatment [8,13,14,15,16,17,18,19,20,21]. However, the KOH treated teeth were dissolved in HCl or HClO_4_, so the whole or part of the specimen was destroyed. It was therefore impossible to determine the change in the amount of fluoride from the superficial surface to the deep area continuously. In this PIXE/PIGE analysis, the tooth specimen did not perish, and the T-F was continuously measured. Thus, our PIXE/PIGE analysis may be useful for measuring T-F or W-F.

At the interface area, PIXE/PIGE analysis revealed significantly different amounts of W-F versus T-F in the CX samples after 7 days. On the other hand, for cumulative values from the interface to 100 µm depth, CX and MB samples on day 7 and all the materials on day 28 showed significantly different levels of W-F versus T-F. Komatsu et al. [22] reported that the amount of fluoride uptake in the enamel and dentin decreased following KOH treatment. Although our results are in line with some aspects of this report, certain differences existed in the materials used and the duration of application between Komatsu et al.’s report and the present study. At the interface area, the fluoride from the materials would be in contact first. The dentin in this area was constantly exposed to fluoride for 28 days; thus, there was sufficient time for conversion of HAP to T-F. On the other hand, for a 100 µm deep area, the distribution of fluoride from materials might take a long time; therefore, T-F would be converted to a small amount. Upon comparison of fluoride release into the water from materials, CX showed the largest amount of fluoride release among all the materials. CX demonstrated significant differences between W-F and T-F under many conditions. A large amount of fluoride was exposed on the dentin surface, and many parts of fluoride could not be converted to T-F, but to CaF_2_-like fluoride, since the amount of fluoride or the ratio of T-F/W-F was inadequate. Since the amount of T-F from the materials seemed to depend on the amount of fluoride uptake into the tooth structure and the duration of fluoride exposure, we also investigated the amount of W-F and T-F in f-HAP to analyze the relationship between T-F and fluoride uptake.

Upon comparing the materials using the PIXE/PIGE analysis, no significantly different values were observed among fluoride-containing materials (CX, FE, and PB) under most conditions. The null hypothesis that a material which releases a lot of fluoride will indicate a lot of tooth-bound fluoride was rejected. The interface between the tooth and the material was covered with wax during the experimental period. Therefore, fluoride transfer (movement) from the material to dentin could be slow since there is nearly no water around this interface. Thus, fluoride distribution from the materials seems to indicate no different values; however, a significant difference was observed in fluoride release from the materials.

Although the amounts of W-F and T-F were higher in f-HAP when the concentration of the fluoride immersion solution was higher, there were no significantly different values between the two duration periods (7 and 28 days), except for T-F following 100 ppmF immersion. Several reports have demonstrated that the T-F levels in enamel and dentin are higher with increased concentrations of applied fluoride [8,16,19,20] and longer application periods [17,18]. However, White et al. [12] reported that the amount of T-F in the enamel slightly increased at low concentrations of applied fluoride. 

In the present HAP study, as the concentration of the fluoride solution increased, the ratio of T-F to W-F tended to decrease for a duration of 7 days. The ratio of T-F/W-F indicates the conversion rate from W-F to T-F; thus, a high ratio indicates that more T-F could be converted from fluoride uptake from material or topical fluoride application. When HAP was immersed in a high concentration of fluoride for short periods, most of the fluoride ions did not replace the hydroxyl ions; thus, it was bound with calcium and other ions. In the 28 days group, there was no significant difference among concentrations because the conversion to T-F might have progressed during this period. A critical fluoride dose exists for the conversion of HAP to fluorapatite. As the application period increased, this conversion continued, so more fluoride ions replaced hydroxyl ions, and more T-F was found.

CX demonstrated a lower ratio of T-F to W-F than FE and PB in the PIXE/PIGE analysis. CX showed the highest fluoride release among the materials used; therefore, the trend of the ratio seems to be similar to that of HAP. Although no reports exist on the ratio of T-F to W-F, S-F decreased and T-F increased over time [13,21]. Thus, it is expected that the ratio seems to be low for short fluoride exposure periods. 

S-F and T-F would be effective for caries prevention as a fluoride provider and acid-resistant layer, respectively. Novel methods for analyzing T-F are revealed; thus, these types of fluoride measuring methods could be explored in more detail. However, the in-air µPIXE/PIGE system is very expensive and requires a huge accelerator, so it is not easy for everyone to use. Furthermore, specimens need to be cut for measurement, and it is not possible to measure the same specimen at different times. Moreover, the conversion of W-F to T-F caused by the fluoride application to the tooth structure means it would be difficult to apply this conversion to clinical use. Nevertheless, the relationships between S-F and tooth demineralization or T-F and tooth demineralization should be considered in future caries prevention studies.

## 5. Conclusions

Our limited study indicates that W-F and T-F in dentin in contact with fluoride-containing coating materials was detected using the in-air micro PIXE/PIGE system. When a high concentration of fluoride solution or high fluoride releasing material was applied to dentin, high W-F and T-F levels were detected. When a low-concentration fluoride solution or low fluoride releasing material was applied to dentin, a high ratio of T-F/W-F was detected.

## Figures and Tables

**Table 1:** Materials used in this study.

Trade Name (Company)	Composition	Code
PRG Barrier Coat (Shofu, Japan)	Base: S-PRG filler, polymeric monomer, water, others active: carboxylic acid monomer, phosphonic acid monomer, polymeric monomer, photo initiator, others	PB
Clinpro XT varnish (3M, USA)	Paste: HEMA, Bis-GMA, initiators, fluoroaluminosilicate glass, water, others Liquid: Polyacrylic acid, water, HEMA, initiators, calcium glycerophosphate, others	CX
Fuji IX EXTRA (GC, Japan)	Powder: Fluoroaluminosilicate glass Liquid: polyacrylic acid, polybasic carboxylic acid, water	FE
Clearfil Mega Bond (Kuraray Noritake Dental, Japan)	Primer: MDP, HEMA, hydrophilic aliphatic dimethacrylate, dl-camphorquinone, water Bond: MDP, Bis-GMA, HEMA, hydrophobic aliphatic dimethacrylate, dl-camphorquinone, initiators, accelerators; silanated colloidal silica	MB

S-SPRG: surface pre-reacted glass ionomer; HEMA: 2-hydroxylethyl methacrylate; Bis-GMA: bisphenol A diglycidyl methacrylate; MDP: 10-methacryloyloxydecyl dihydrogen phosphate.

**Table 2:** Ratio of tooth-bound fluoride (T-F)/total fluoride (W-F) on the interface area and cumulative values to 100 µm depth (n = 6 in each material).

Material	7 Days Interface	7 Days Cumulative	28 Days Interface	28 Days Cumulative
CX	0.40 (0.01–0.97) ^a^	0.07 (0.00–0.21)	0.32 (0.00–0.88) ^a^	0.24 (0.02–0.51) ^a^
FE	0.58 (0.09–0.87) ^a^	0.61 (0.07–0.86) ^a^	0.65 (0.06–0.93) ^a^	0.58 (0.06–0.80) ^a^
PB	0.43 (0.00–0.93) ^a^	0.49 (0.20–0.89) ^a^	0.65 (0.09–0.86) ^a^	0.43 (0.13–0.60) ^a^

Values are shown as median (lowest–highest). The same letter in each column indicates no significant difference (*p* > 0.05).

**Table 3:** Amounts of total fluoride content (W-F), tooth-bound fluoride (T-F), and the ratio of T-F/W-F on fluoride-treated hydroxyapatite; n = 9.

Conc.	Period	W-F		T-F		Ratio (T-F/W-F)	
1 ppmF	7 days	2.65 (0.04)	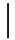	2.55 (0.07)	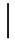	0.66 (0.03) ^a^	
	28 days	2.41 (0.23) *		2.26 (0.59) *		0.87 (0.08) ^b^	
10 ppmF	7 days	16.9 (1.07)	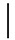	14.9 (0.91)	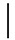	0.88 (0.06) ^a^	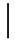
	28 days	19.1 (1.49)		17.7 (1.35)		0.92 (0.08) ^b^	
100 ppmF	7 days	157 (8.59)	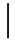	104 (4.51)		0.96 (0.02)	
	28 days	152 (12.5)		132 (10.1)		0.82 (0.13) ^b^	

Values on W-F and T-F shown as mean (standard deviation) in µmol of fluoride/mg of hydroxyapatite. Asterisks (*) indicate no significant difference between values on W-F and T-F in each row (*p* > 0.05). Bars connected values indicate no significant difference between on two different duration periods (*p* > 0.05). Same letters in each duration period indicate no significant difference between concentrations (*p* > 0.05).

## Data Availability

Not applicable.
